# A sport room within the paediatric oncology ward

**DOI:** 10.3332/ecancer.2021.ed108

**Published:** 2021-02-05

**Authors:** Gabriel Revon-Rivière, Paul Saultier, Rova Andrianarivony, Clothilde Vallet, Nicolas André

**Affiliations:** 1 APHM, La Timone Children’s Hospital, Department of pediatric hematology, immunology and oncology, Marseille, France; 2 Aix Marseille Univ, INSERM, INRAe, C2VN, Marseille, France; 3 Service de pediatrie, CHU Mère Enfant Tsaralalàna, Antananarivo, Madagascar; 4 Sourire à la Vie, Marseille France; 5 SMARTc unit Centre de Recherche en Cancérologie de Marseille Inserm U1068 Aix Marseille University, Marseille, France.

**Keywords:** physical activity, child, cancer, sports, physical activity, exercise tolerance, quality of life

## Abstract

There is a growing interest in physical activity (PA) in paediatric oncology. Overall studies in children with cancer have reported good adherence, positive trends in health status, and no adverse events. Thus, a general PA program should be offered to paediatric oncology inpatients. Anyhow, the absence of a dedicated place to perform PA sessions beyond the paediatric oncology department corridors and patients' room has been identified as one of the major limiting factors. We do believe "in the ward" sport rooms should be further implemented and evaluated in paediatric oncology departments worldwide.

Over the last decade, a growing interest in physical activity (PA) as a tool to improve health and to help fight diseases has increased [1], notably in the oncology community. Indeed, an analysis of almost 1.5 million adults has shown that leisure-time PA of a moderate to vigorous intensity was associated with a reduced risk of occurrence for at least 13 types of adult cancer [2].

A similar trend is also observed in pediatric oncology. Thus, using the search criterias “physical activity”, “sport”, “children” and “cancer” a pubmed survey shows a marked increase in the number of publications on this topic from about 10 publications per year before 2010 to more than 80 publications per year in 2019. Over the last two years, over 140 papers have been published. Among these, only 60 actually specifically deal with PA and children with cancer. Interestingly, the large majority are investigational studies (n=47) followed by reviews (n=8) and letters or editorials (n=4) illustrating the need to generate new clinical data.

While the effects of PA on adult cancer patients are being increasingly characterized [3], data on effects of PA in pediatric oncology patients remain scarce. A recent systematic review of the literature identified 10 studies, involving 204 patients [4]. Overall, good adherence, positive trends in health status, and no adverse events were noted. Common strategies included individualized, closely supervised, combination training with adaptability to meet fluctuating patient abilities depending on their treatment types and phases. Based on their analysis, the authors recommended that a general PA program should be offered to pediatric oncology inpatients. Nevertheless, randomized trials demonstrating the benefit of PA and providing high-level evidence in this context are so far still limited.

We recently published a randomized trial evaluating the safety and efficacy of a PA program in children and adolescents with cancer [5]. Patients were randomly assigned in a 1:1 ratio to a 6-month PA program or to the control group. Eighty children and adolescents (age range 5.0-18.4 years) were included. Underlying malignancies were leukemia (39%) and a broad range of solid tumors (61%). No adverse event occured. At 6 months, the evolution of the 6 minutes walking test distance was significantly improved in the intervention group versus the control group (86±12m vs. 32±6m; p<0.001). Several other physical parameters were significantly improved in the intervention group. Global self-esteem and parent-reported QoL were also significantly increased in the intervention group. This study shows that in children and adolescents with cancer, an adapted PA program is safe, can improve exercise capacity and may have physical and psychological benefits [5].

We do believe beyond the results of this randomized trial, a crucial point is the availability of a dedicated gym within the pediatric oncology ward. Indeed, a recent survey among 34 centers of the SFCE (Société Francaise de lute contre les Cancers de l’Enfant) has identified many barriers to the practice of PA for children with cancer in France [6]. The absence of dedicated place to perform PA sessions beyond the pediatric oncology department corridors and patients’ room was identified as one of the major limiting factors.

In collaboration with a nonprofit organization (Sourire à La Vie; https://www.sourirealavie.fr), our pediatric oncology department has implemented a genuine gym located within the ward itself. The room is supervised by trained PA intructors and welcome all children aged >4 years old, treated in the department during their stay in the day care unit or hospitalization. PA is proposed after validation by a senior pediatric oncologist. This room is equipped with classical muscular strengthening equipment such as balance platforms, dumbbells, elastic draw, jump ropes, medicine ball, gym ball, and step. It also provides a basketball hoop, table tennis, badminton net and other racket games support (i.e. played with badminton rackets and balloons), darts, archery material, boxing gloves and shields, soccer ball and goal, music for dancing, a treadmill with virtual scenery (Technogym, Spazio Forma), a bike with virtual scenery (Proform, bike TDF pro 5.0) and active video game consoles (Wii or the Xbox 360 with Kinect). The Sport room is divided in different spaces to allow concurrent activities with different children and adolescents. PA sessions can be individual or collective but with no more than 4 children at the same time. The close proximity with the nurses allows its use even in children receiving chemotherapy, hydration, or blood transfusion. Monitoring gym use during the opening year showed that 107 patients used it (out of 150 new patients with cancer per year in our department) over 200 days, with a median number of PA sessions per patient of nine. Overall, 1.000 hours of PA were provided during the first year and thanks to close interactions between physician and PA specialists no sides effects of PA were reported.

While evidence accumulates to better delineate the benefits of PA for children with cancer, how to provide it, and how to use it for anticancer purposes [7] it seems that PA should be used even in the initial sometimes intensive phase of treatment. In this case, performing PA not only in the room of the patients, the corridors of the department, or distant gymnasium but within the pediatric room provides a unique opportunity to increase the number of hours of PA that can be performed by children. We do believe this should be further implemented and evaluated in pediatric oncology wards worldwide.

## Conflicts of interest

The authors have no conflicts of interest to declare.

## Funding

No direct funding for this article was received.
